# Treatment of Hepatitis C Virus Infection and Associated Vascular Complications: A Literature Review

**Published:** 2014-05

**Authors:** Reza Karbasi-Afshar

**Affiliations:** Atherosclerosis Research Center, Baqiyatallah University of Medical Sciences, Tehran, Iran

**Keywords:** Hepatitis C virus, HCV, Vascular disease, Heart, Risk factor

## Abstract

Interferon (IFN)-based therapy, the cornerstone for treatment of hepatitis C virus (HCV) infection, is generally considered to be the single most effective treatment strategy for this infection. Although most adverse effects of IFN therapy respond very well to the cessation of this drug, there are reports of serious irreversible adverse effects. This review article evaluates the adverse effects of IFN therapy in HCV-infected patients. We have undertaken an extensive search for articles regarding IFN and pegylated-IFN (PEG-IFN) therapy and their vascular complications using multiple sources that include PubMed, publishers’ websites, and Google Scholar. The prevalence of ocular disorders in the early period (first 8 weeks) after IFN administration was high with over half of the patients experiencing these adverse effects. Several authors strongly propose screening programs for retinopathy in the early period after IFN administration. Pulmonary hypertension due to IFN therapy is a serious side effect due to its irreversible nature in most patients. Patients who develop signs of acute abdomen up to months after IFN administration should be rapidly assessed for potential adverse effects of IFN. The literature suggests a broad spectrum of vascular injuries to different organs in humans as adverse effects of IFN therapy in HCV-infected patients.

## Introduction

Interferon (IFN) is a cytokine broadly used to treat viral infections, malignancies, and disorders of the immune system. IFN is highly effective in the treatment of tumors that affect the vascular system, including hypernephroma,^1^ hemangiomatosis^2^ and gastrointestinal tumors,^3^ in which significant vascularization develops. In the context of gastroenterology, the significance of IFN is mainly due to its prominent role in the treatment of chronic active hepatitis in patients infected with hepatitis C virus (HCV) and/or hepatitis B virus. IFN-based therapy is the cornerstone and one of the most effective treatment strategies for HCV infection. However, despite the mentioned advantages, IFN administration is accompanied by complications that affect various organs and systems, necessitating serious consideration prior to its administration. The relevance of this issue is underscored when one considers that IFN-associated adverse effects can result in the discontinuation of treatment.^4^ A recent series has reported the discontinuation of IFN in 17% of HCV-infected patients, mainly due to cardiovascular complications.^5^ Apart from one report from a large population, much lower frequencies of cardiovascular complications of IFN [7 out of 11241 (0.06%)] in patients with chronic hepatitis have been reported.^6^

The cardiovascular complications of IFN therapy include pericarditis, rhythm disturbances, myocarditis, cardiomyopathy, congestive heart failure, vasospastic angina pectoris, microvascular angina, peripheral vascular disorders, stroke, and myocardial infarction. These complications have been reported as case reports and series from different centers; however, to the best of our knowledge there is no article that has comprehensively reviewed the existing data to provide clinicians with an inclusive view on this subject. HCV-infected patients are mostly complicated with several other disorders, and a cardiovascular complication may not be considered a side effect to IFN therapy. Therefore, this review article aims to categorize different cardiovascular complications associated with IFN therapy in HCV-infected patients and to provide sufficient information for future planning in order to ensure higher safety for HCV-infected patients who undergo IFN therapy.

## Literature Review

We undertook an extensive search for articles that concerned IFN and PEG-IFN therapy and their vascular complications by using multiple sources including PubMed, publishers’ websites, and Google Scholar. We assessed all articles for the levels of evidence and employed the following keywords or combinations thereof: interferon; pegylated interferon; hepatitis C virus; HCV; cardiovascular complication; heart; coronary artery; cardiomyopathy; pericarditis; myocarditis; retina; retinopathy; nephropathy; pulmonary; lung disease; gastrointestinal; intestinal injury; myopathy; myositis; myopathy; ischemia; thyroiditis; etc. 

## Arrhythmias after Interferon (IFN) Therapy

IFN therapy for HCV infection has been associated with myocardial function disorders.^7^^,^^8^ Takase et al.,^9^ followed 9 HCV-infected patients undergoing a course of recombinant IFN therapy. These authors reported that INF treatment resulted in decreased exercise tolerance time from 449±94 s to 329±67 s (P<0.05) and decreases in several heart rate variability parameters [S.D. index, 42±5 ms vs. 37±9 ms, root mean square successive difference (rMSSD), 22±5 ms vs. 19±4 ms, >50 ms differences between adjacent NN (pNN50), 4±3% vs. 2±1%; P<0.05]. Several reports indicated an association between IFN therapy in HCV-infected patients to arrhythmias, either directly or secondary to endocrine or pulmonary system disturbances. Hiramatsu et al.,^10^ in a cohort of 22 patients, suggested that arrhythmias which developed as a consequence of IFN therapy in patients with active hepatitis were reversible and subclinical with no sustained ventricular arrhythmia detected throughout the follow-up period. However, their cohort consisted of a limited number of patients and serious arrhythmias could occur occasionally. Teragawa et al.^11^ studied 295 HCV-infected patients during their treatment course of IFN therapy and after one year. They found that 4 (1.4%) patients developed arrhythmias; this was only 40% of the overall cardiovascular complications of HCV treatment. Fujiwara et al.^12^ reported the case of a 64-year-old man infected with HCV. Seven days after starting IFN, the patient developed a giant T wave inversion visualized on a check-up electrocardiogram (ECG). In addition, ten days after IFN administration the patient’s clinical symptoms included fatigue, palpitations, a depressive feeling, tachycardia of 100 beats/min, supraventricular premature beats, atrial fibrillation, and septal and apical hypertrophy. At four days after cessation of IFN therapy the patient’s subjective symptoms improved and atrial fibrillation disappeared, however his giant T wave inversion and apical hypertrophy remained detectable several months after the discontinuation of the drug. Another case report from Poland indicated atrioventricular (AV) conduction disturbances in the form of a second-degree AV block in a 55-year-old woman with no known cardiac disorders prior to treatment with pegylated IFN (PEG-IFN) therapy for an HCV infection.^13^ Drug cessation resulted in a significant drop in the electrocardiographic disturbances. These cases have shown that although the discontinuation of PEG-IFN can revert some arrhythmic changes, however others are likely to remain. In hemophilic patients simultaneously infected with HCV and HIV, therapy with IFN-alpha-2a has been associated with a 14% incident rate of tachycardia, leading to a decrease in the administered IFN dose.^14^

Torsades de pointes,^15^ sinus bradycardia,^16^^,^^17^ transient sinus tachycardia and premature ventricular beats^18^ have been observed in HCV-infected patients undergoing IFN therapy. They have occasionally resulted in the cessation or dose reduction of the drug. There are also reports indicative of thyroiditis and associated arrhythmias, especially tachycardia, after the administration of IFN-alpha in HCV-infected patients; this issue has been very well reviewed by Menconi et al.^19^

## Pericarditis and Myocarditis after Interferon (IFN) Therapy in HCV-Infected Patients

Teragawa et al.^11^ reported a case of pericarditis that developed in an HCV-infected individual on IFN therapy. Since then, several other studies have reported similar cases.^20^^,^^21^ Boonen et al.^22^ reported a 24-year-old woman that underwent IFN-alpha therapy for HCV infection and subsequently developed pericarditis; later tests showed that she all criteria necessary for the diagnosis of systemic lupus erythematosus (SLE). The authors concluded that IFN-alpha therapy could result in the provocation of autoimmunity and they supported their hypothesis by citing several reports of other autoimmune disorders which occurred after IFN therapy in HCV-infected patients (including Grave’s disease and sarcoidosis). Another case-report study reported similar adverse effects for PEG-IFN-2a in a 67-year-old male, in whom discontinuation of the drug plus Prednisolone therapy cured pericardial effusion within 16 days.^23^ An immune reaction might partially explain the rationale behind the pericardial effusion and pericarditis that developed after IFN therapy in HCV-infected patients.^22^ In these cases corticosteroid therapy has been highly recommended.^23^

PEG-IFN has been reported as a precursor for acute pericarditis that developed in HCV-infected patients seven months after starting PEG-IFN, which resolved following complete cessation of the drug.^24^ In hemodialysis patients with HCV infection PEG-IFN resulted in pericarditis two weeks after administration, which necessitated discontinuation of treatment.^25^ On the other hand, HCV infection itself has been a known factor for the development of pericarditis and PEG-IFN therapy has been used to treat the associated pericarditis.^26^ Myocarditis is a more serious side effect of IFN therapy that can result in cardiogenic shock^11^ and/or fatal consequences in HCV-infected patients.^27^ As is the case in pericarditis, HCV infection can also give rise to myocarditis and for its treatment, IFN therapy has been successfully employed.^28^^,^^29^ This controversy is of utmost importance. Physicians should determine whether the injury is due to an HCV infection or IFN therapy-this will have a decisive effect on the therapeutic approach. If the problem is due to an HCV infection, IFN should be administered or the dose augmented. However if it is an adverse effect of IFN, drug administration should be discontinued. Some authors have proposed that in cases where pericarditis is considered as a manifestation of HCV-induced autoimmunity, administration of IFN is contraindicated because it can exacerbate the disease.^30^^,^^31^

## Acute Coronary Syndrome and Interferon (IFN) Therapy in HCV Infection

Acute coronary syndromes are among the rare adverse effects of HCV infection therapy^32^ that are probably attributed to IFN-induced vasospasms.^33^ The most notorious side effect of Ribavirin is hemolysis; the stress of a sudden onset of anemia due to hemolysis can induce myocardial infarction in persons with pre-existing coronary artery disease or stroke in those with cerebrovascular disease. This is the only cardiovascular side effect usually attributed to Ribavirin, one of the key agents in HCV combination therapy.^32^ Shakil et al.,^34^ in a follow-up of 38 HCV-infected liver transplant patients, introduced 2 patients who experienced myocardial infarctions after starting IFN plus Ribavirin therapy, both had normal cardiovascular evaluations prior to the study onset. The first patient was a 53-year-old man who developed myocardial infarction at week 28 and died with normal hemoglobin levels. The second, a 39-year-old woman, developed a myocardial infarction at week 48 concomitant with a sharp decline in the hemoglobin level from 13.1 g/dl to 8.7 g/dl; she survived the myocardial infarction after a reduction in the drug dose.^34^ IFN alone has been shown to cause myocardial infarctions in HCV-infected patients.^35^^,^^36^ IFN cardiotoxicity may be related to the febrile reaction induced by exposure to IFN therapy, inducing an increase in oxygen demand and resulting in an acute coronary syndrome attack such as a myocardial infarction.^37^ Therefore, any acute coronary event after the initiation of IFN and Ribavirin in an HCV-infected patient should alert physicians and modifications in IFN and Ribavirin dosage administration should be seriously considered.

## Cardiomyopathy

HCV infection has been reportedly associated with the development of cardiomyopathy. IFN therapy has been suggested for the treatment of HCV-related cardiomyopathy.^29^ One study reported improved serum levels of creatine kinase (CK), CK-MB, and cardiac troponin T during treatment with IFN whereas the conventional treatment for heart failure was not effective.^38^ On the other hand, some authors, including Dalekos et al.^39^ and Kuethe et al.,^40^ found no association between cardiomyopathy and HCV infection.

The development of cardiomyopathy in HCV-infected patients, due to the above-mentioned evidence, may inevitably lead to a diagnosis of HCV-related cardiomyopathy, whereas none of the above mentioned articles have discussed a history of IFN therapy. As a result, one may assume that at least some of those cases might have developed cardiomyopathy as an adverse effect of anti-HCV therapy rather than the HCV infection itself.^41^

IFN therapy has been reported to induce hypertrophy of the septal and posterior wall of the left ventricle days after the onset of treatment.^42^ There is a report of the development of fatal cardiomyopathy due to IFN therapy in an HCV-infected patient.^43^

Mateo et al.^44^ reported a male liver transplant recipient who underwent IFN therapy for allograft dysfunction due to HCV reactivation. At the commencement of treatment, the patient developed weakness and malaise that necessitated a reduction in drug dosage. The patient, 11 days after discharge and following a transfusion of packed red blood cells became critically ill with a T-wave inversion in his ECG; ejection fraction of 28%; and dilated cardiomyopathy in the thoracic ECG. Discontinuation of IFN improved his symptoms, and subsequently IFN was readministered with a gradual increase in dosage. Not only does this interesting case show the reversibility of IFN-induced cardiomyopathy but it has demonstrated that after cessation of IFN therapy and treatment of the primary reaction, with caution IFN can be safely re-administered. There are reports that the cardiotoxicity of IFN therapy can be irreversible. Dhillon et al.^45^ have reported irreversible cases of pulmonary hypertension induced by IFN therapy in HCV-infected patients. The next section presents an in-depth discussion on such cases.

## Microvascular Injury to the Heart, Kidneys, and Retina after Interferon (IFN) Therapy

It has been demonstrated that IFN can aggregate leukocytes within the capillaries, resulting in vascular damage and micro-infarctions. This can affect a number of organs, particularly the heart and eyes. In a mouse model, Salman et al.^46^ have reported that IFN-alpha-2b therapy in mice could lead to a significant increase in the thickness of the endothelial processes of the myocardial capillary walls, with a subsequent decrease in the size of the capillary lumen. This might explain the augmented rate of cardiomyopathies, especially dilated cardiomyopathy, in HCV-infected patients who undergo IFN therapy.

Cardiomyopathy is not the only IFN-induced complication as an adverse effect for IFN therapy in HCV-infected patients. Retinopathy is one of the most frequently reported microvascular complications of IFN in HCV-infected individuals. Both arterial and venous origins have been proposed as culprits. In some cases, the retinopathy is irreversible. In a prospective cohort of 63 patients with chronic HCV infection and no retinal lesions before IFN-alpha therapy, Kawano et al.^47^ reported that over 57% of the patients developed retinopathies, including 25 (40%) cases of retinal hemorrhage and 28 (44%) cases of cotton wool spots. Interestingly, 86% of the lesions were diagnosed within 8 weeks after onset of IFN administration. In a similar study by Sugano et al.,^48^ 24% of the HCV-infected patients who started IFN therapy developed retinal hemorrhage, with two-thirds of them developing cotton wool spots and half having visual symptoms. Consistent with the previous study, approximately 85% of the patients developed their retinal signs just within the first 8 weeks post-IFN administration. In a larger recently published study, Vujosevic et al.^49^ demonstrated that 31% of the HCV-infected patients undergoing PEG-IFN and Ribavirin developed retinopathy. Hypertension was defined as the only independent factor associated with IFN-induced retinopathy [hazard ratio (HR)=4.99, 95% confidence interval (CI): 2.29-10.89]. Only one (1.1%) patient needed therapy cessation due to bilateral branch retinal vein occlusion. The authors conducted a cost-effective study and showed that screening for PEG-IFN induced retinopathy was cost-effective.

## Pulmonary Injury due to Interferon (IFN) Therapy in HCV Infection

Pulmonary dysfunction due to IFN therapy in HCV-infected individuals has been reported. Garib et al.^50^ reported dyspnea after PEG-IFN therapy in HCV patients with advanced liver disease. Spirometric changes in FEV1 and FEV1/FVC were also observed. Kumar et al.^51^ introduced 4 patients who developed significant pulmonary signs and symptoms while they were on therapy with IFN-alpha and Ribavirin for chronic HCV; final diagnosis was bronchiolitis obliterans organizing pneumonia in 2 patients, and interstitial pneumonitis in 2 others. In all patients, pulmonary symptoms resolved after cessation of the drug.

Pulmonary hypertension is another adverse effect of IFN therapy in HCV-infected patients and has been repeatedly reported by different authors. Dhillon et al.^45^ reported 4 cases of irreversible pulmonary hypertension that developed after IFN therapy in HCV-infected patients. One of the patients was a 35-year-old male liver transplant recipient who developed pulmonary hypertension after IFN therapy for HCV and died about 2.5 years later, with no response to treatments. The second was a 40-year-old female HCV-infected individual who developed pulmonary hypertension 32 months after IFN therapy. Although the treatments failed to reverse her hypertension, she was stabilized. The third patient was a 50-year-old male liver transplant recipient who developed pulmonary hypertension after 10 months of IFN therapy. Although his hypertension did not respond to therapy, his clinical condition stabilized. The fourth patient was a 49-year-old male HCV-infected person who developed pulmonary hypertension 8 months after IFN initiation. The therapy was not successful in treating the hypertension, however the patient was stabilized.******

## Ischemia in the Gastrointestinal Tract

Ischemia in the gastrointestinal tract is another peripheral vascular disorder associated with IFN therapy in HCV-infected patients.^52^ Leung et al.^53^ have reported an interesting case of ischemic colitis that developed 34 weeks after PEG-IFN-alpha; the patient’s symptoms rapidly resolved with the cessation of therapy. Similar observations were reported by Tada et al.,^54^ who presented 2 cases of IFN-alpha-induced ischemic colitis that developed 2 and 6 months after IFN administration. Both cases had complete resolution two weeks after cessation of IFN therapy. A recent case report by Baik et al.^55^ showed the development of ischemic colitis 19 weeks after the commencement of PEG-IFN-alpha-2a and Ribavirin therapy; the case was thoroughly cured only with cessation of the drug. However, not all cases of ischemia in the gastrointestinal tract had benign courses. Pompili et al.^56^ introduced a 53-year-old male with HCV infection who developed jejunal ischemia 3 months after the initiation of therapy with PEG-IFN. Although the patient underwent emergent jejuna resection, he died 6 months after surgery due to preoperative complications. A case of phlegmonous colitis that developed in a chronic HCV-infected patient who received combined therapy of PEG-IFN and Ribavirin also had a lethal consequence.^57^ A case of neutropenic enterocolitis that developed 22 weeks after the initiation of PEG-IFN-alpha-2a and Ribavirin had a fatal outcome.^58^

## Other Systemic Complications Induced by Interferon (IFN) Therapy

Wang et al.^59^ reported a case of acute heart allograft failure induced by PEG-IFN-alpha-2b therapy due to HCV infection in a 50-year-old male which proved fatal. Postmortem autopsy showed no evidence of cellular or humoral rejection, which confirmed that the case was a fatal cardiotoxicity adverse effect due to Peg-IFN-alpha-2b.

Myopathy and acute myositis have also been reported as adverse effects of PEG-IFN-alpha-2b therapy in HCV-infected patients. This may contribute, in part, to the cardiovascular disorders that are seen in these patients. Venezia et al.^60^ have reported a case of acute myositis after PEG-IFN-alpha-2b therapy for an HCV infection and suggested that a rapid discontinuation of IFN could resolve the problem in such cases. Golstein et al.^61^ reported a reversible case of myopathy that developed as a side effect of the same agent, which improved after drug cessation.

Disorders of the immune system have also been reported as a consequence of IFN therapy in HCV-infected patients. The importance of these diseases is attributed to their direct or indirect effects on the cardiovascular system. Sarcoidosis is one of the most prevalently reported immune system disorders.^62^ SLE has also been reported as an adverse effect of IFN therapy in HCV-infected patients.^63^ Cryoglobulinemia is another immune disorder that has been reportedly associated with IFN therapy.^64^ Type I diabetes mellitus, another autoimmune disorder, has been repeatedly suggested to develop as an adverse effect of IFN therapy.^65^ Grave’s disease and thyroiditis are other disorders attributed to IFN therapy in HCV-infected patients.^66^^,^^67^ ******

## Conclusion

IFN-based regimens are a cornerstone for the treatment of HCV infection and generally considered effective as treatment for this infection. Although most adverse effects of IFN therapy respond very well to cessation of the drug, this review article shows that there are serious adverse effects associated with IFN therapy in HCV-infected patients, which might affect sensitive organs such as the eyes, heart, and lungs. The prevalence of ocular disorders in the early period (first 8 weeks) after IFN administration can amount to 57% and result in irreversible, significant damage to visual acuity. Pulmonary hypertension secondary to IFN therapy is a serious side effect due to its irreversible nature in most patients and as such necessitates prompt evaluation. After mentioning these adverse effects of IFN therapy in HCV patients, it should be mentioned that HCV infection itself may produce cardiovascular injuries. Only a few of the articles we have reviewed were case-controls, thus we cannot separate the potential effects of HCV from IFN therapy adverse effects. More prospective studies should be conducted for this purpose. To the best of our knowledge, there is no study in the current literature that concerns prevention strategies for the vascular effects of IFN therapy on different organs. We suggest that prospective studies be undertaken to address this critical issue. 
